# The role of HOTAIR in the modulation of resistance to anticancer therapy

**DOI:** 10.3389/fmolb.2024.1414651

**Published:** 2024-06-03

**Authors:** Monica Cantile, Valentina Belli, Giosuè Scognamiglio, Anna Martorana, Giovanna De Pietro, Maura Tracey, Alfredo Budillon

**Affiliations:** ^1^ Scientific Directorate, Istituto Nazionale Tumori IRCCS Fondazione G. Pascale, Naples, Italy; ^2^ Rehabilitation Medicine Unit, Istituto Nazionale Tumori IRCCS Fondazione G. Pascale, Naples, Italy

**Keywords:** long non-coding RNAs, resistance to anti-cancer therapies, HOTAIR in drugresistance, HOTAIR as circulating biomarker, HOTAIR as therapeutic target

## Abstract

Leading anti-tumour therapeutic strategies typically involve surgery and radiotherapy for locally advanced (non-metastatic) cancers, while hormone therapy, chemotherapy, and molecular targeted therapy are the current treatment options for metastatic cancer. Despite the initially high sensitivity rate to anticancer therapies, a large number of patients develop resistance, leading to a poor prognosis. The mechanisms related to drug resistance are highly complex, and long non-coding RNAs appear to play a crucial role in these processes. Among these, the lncRNA homeobox transcript antisense intergenic RNA (HOTAIR), widely implicated in cancer initiation and progression, likewise plays a significant role in anticancer drug resistance. It can modulate cell activities such as proliferation, apoptosis, hypoxia, autophagy, as well as epithelial-mesenchymal transition, thereby contributing to the development of resistant tumour cells. In this manuscript, we describe different mechanisms of antitumor drug resistance in which HOTAIR is involved and suggest its potential as a therapeutic predictive biomarker for the management of cancer patients.

## Introduction

The major anticancer therapies include surgery, radiotherapy, endocrine therapy, chemotherapy, molecular targeted therapy, and immunotherapy, used as monotherapy and in combination with each other (Pérez-Herrero and Fernández-Medarde, 2015; Kruger et al., 2019; Bedard et al., 2020; Kareliotis et al., 2020; Jin, Wang and Bernards, 2023). However, anticancer drug resistance represents a major clinical challenge and one of the main causes of poor prognosis, tumour metastasis and recurrence (Kalbasi and Ribas, 2020; Varshney et al., 2023). Therapeutic resistance is a complex process that can arise from genetic, epigenetic, and microenvironmental factors. It can be categorized into intrinsic or primary resistance, due to endogenous tumour cell factors which provide survival advantages and adaptability to therapy, and acquired or secondary resistance (Wicki et al., 2016). Most studies have primarily focused on understanding the mechanism of acquired drug resistance. Due to heterogeneous tumour development, minimal residual tumour cells may indeed adopt mutationally dependent or independent resistance to the drug (Lim and Ma, 2019). Resistant cancer cells often exhibit cross-resistance to different anticancer drugs, through a phenomenon known as multidrug resistance (MDR), making resistance mechanisms not mutually exclusive (Assaraf et al., 2019; Bukowski, Kciuk and Kontek, 2020). Drug resistance occurs simultaneously or sequentially during therapy which suggests the need to implement combined anti-cancer treatments as well as identification of molecular mechanisms capable of simultaneously regulating multiple processes related to resistance (Chatterjee and Bivona, 2019; Meador and Hata, 2020).

The main mechanisms related to anti-drug resistance include: 1) apoptosis suppression; 2) modification of drug metabolism (reduction of intracellular drug deposit by decreased uptake or increased efflux); 3) alteration of drug targets; 4) promotion of pathways for cell proliferation and survival; 5) regulation of DNA repair processes; 6) induction of epithelial-mesenchymal transition (EMT); 7) alteration of tumour microenvironment (TME); 8) promotion of autophagy; ix) different epigenetic events, including methylation of DNA, histone alterations as well as aberrant expression of non-coding RNAs (ncRNAs) (Bukowski, Kciuk and Kontek, 2020; Haider et al., 2020; Nussinov, Tsai and Jang, 2021).

NcRNAs are molecules that have no protein-coding functions and comprise microRNA (miRNA) and long ncRNAs (lncRNAs). They are involved in many physiologic cell processes, such as chromatin modification, gene transcription, post-transcriptional regulation and modulation of different signal transduction pathways (Esteller, 2011; Kung, Colognori and Lee, 2013; Ha and Kim, 2014; Statello et al., 2021; Virciglio, Abel and Rederstorff, 2021). They are also crucial mediators of carcinogenesis as well as tumour progression which play a crucial role in resistance to anticancer drugs by targeting many oncogenes and oncosuppressor genes (Lee and Dutta, 2009; Gibb, Brown and Lam, 2011; Matsui and Corey, 2017). In particular, lncRNAs have been considered fundamental modulators of chemoresistance, influencing drug metabolism, apoptosis, DNA repair, and drug targets alteration (Mahinfar et al., 2021; Ye et al., 2022). Among them, HOTAIR plays a critical role in both the initiation and progression of different types of human cancers, as well as in the modulation of resistance mechanisms, facilitated also by its functional interactions with different miRNAs (Bhan and Mandal, 2015; Cantile et al., 2021; Liguori et al., 2021; Xin et al., 2021).

In this review, we focus on the role of the lncRNA HOTAIR in the mechanisms of resistance to the main anticancer therapies and discuss its potential as a predictive biomarker in diagnostics and management of cancer patients.

### The role of HOTAIR in cancer

The lncRNA HOTAIR, located between HOXC11 and HOXC12 genes on chromosome 12q13.13 (Rinn et al., 2007), plays a crucial role as a modulator of chromatin remodelling and transcriptional silencing (Bhan and Mandal, 2015). Majello and colleagues demonstrated that HOTAIR functions as a molecular scaffold, capable of binding Polycomb repressive complex (PRC2) at the 5′end, inducing trimethylation of histone complex H3K27, and to the lysine-specific histone demethylase 1A (LSD1) at the 3′end, thereby contributing to gene silencing (Majello et al., 2019). However, other studies have shown conflicting conclusions regarding the direct interaction between lncRNAs and PRC2 evidencing that the first proposed model remains disparate (Kaneko et al., 2014; Beltran et al., 2019; Song et al., 2023). Among those, Portoso et al. demonstrated that artificially tethering HOTAIR to chromatin resulted in transcriptional repression, and this effect did not require PRC2. Instead, PRC2 recruitment seemed to be a consequence of gene silencing (Portoso et al., 2017). In addition, Guo et al. showed that PRC2 and other chromatin proteins do not appear to directly bind to RNA *in vivo*, without excluding the possibility that they can bind to specific RNAs through other mechanisms not yet known, or by binding to RNA indirectly through protein-protein interactions (Guo et al., 2024). HOTAIR regulates various physiological cellular processes associated with cell cycle progression, by modulating cell cycle-related protein expressions and inducing cell cycle arrest at the G0/G1 phase (Zhang et al., 2013). Furthermore, HOTAIR can regulate various cell functions by acting as a sponge for multiple miRNAs, thereby reducing their expression and increasing the expression of numerous miRNA targets, including both oncogenes and tumour suppression genes (Qiu et al., 2022; Huang and Xiang, 2023; Jiang, Xu and Ren, 2023; Le et al., 2023; Liu et al., 2023; Chen et al., 2024). Some miRNAs would, in turn, be capable of binding HOTAIR in a sequence-specific manner and suppressing its expression and functions (Chiyomaru et al., 2014).

The deregulation of HOTAIR is strongly related to cancer development and progression (Table 1) (Zhang et al., 2014; Liguori et al., 2021; Xin et al., 2021; Qu et al., 2023; Tufail, 2023; Zhou et al., 2023; Hakami et al., 2024).

**TABLE 1:** Summary of the role of HOTAIR in the most frequent tumor types

Tumor type	Function	miRNAs interaction	Circulating marker	References
Breast cancer	ER pathway activation, metastasis promotion, autophagy and EMT	miR130a, miR148a, miR218, miR-130b-3p, miR-449b-5p	blood, serum, plasma	Bhan et al., 2014, Abdel-Hamid NR et al., 2023, M. Zhang et al., 2021b, Hu et al., 2019, Shuqin Zhang et al., 2020d
Prostate cancer	AR stabilization, cell growth and invasion	miR-590-5p		Zhang et al., 2015, Wang et al., 2020
Lung cancer	Migration, invasion, proliferation	miR-217, miR-326	plasma	S.-S. Chen et al., 2019b, Li et al., 2016, Fang et al., 2016; Li N, et al., 2017
Colorectal cancer	Proliferation, Invasion, migration metastasis promotion	miR-34a miR-93	plasma	Tatangelo et al., 2018, Peng et al., 2019, Yang et al., 2016, Liu et al., 2020
Gastric cancer	Migration, invasion, proliferation, and apoptosis reduction	miR-126 miR-195-5p miR-217 miR203a-3p	serum	Jie Zhang et al., 2020a; P. Chen et al., 2023a, Yan et al., 2016, Luo et al., 2023, H. Wang et al., 2018a, Xiao et al., 2018
Ovarian cancer	Proliferation, Invasion, migration metastasis promotion	miR-200c, miR-206, miR-222-3p miR138-5p	plasma	Qiu et al., 2015, Yang C, 2020, Chang L, 2018, Fan L et al., 2022, Yun Zhang et al., 2020e
Cervical cancer	Proliferation, Invasion, migration metastasis promotion	miR-26b		He et al., 2014, Huang et al., 2014, Zhang et al., 2022
Oral cancer	Proliferation, Invasion, migration metastasis promotion	miR-326 miR-613106a-5p		Tao et al., 2020, X Wang et al., 2018b, Zhou et al., 2018, Cao et al., 2022
Pancreatic cancer	Migration, invasion, proliferation, and apoptosis reduction	miR-613, miR-34a,	saliva	Cai H et al., 2017, Deng S et al., 2021, Xie et al., 2016
Bladder cancer	Migration, invasion, proliferation	miR-205	urine	Sun X et al., 2015, Berrondo et al., 2016
Melanoma	Metastasis promotion	miR-152-3p	serum	Cantile et al., 2017, Luan W et al., 2017

It is capable of modulating not only the cell cycle but also cell metabolism, EMT, autophagy, self-renewal and metastatic switch (Li et al., 2014; Pawłowska, Szczepanska and Blasiak, 2017). The role of HOTAIR in tumour behaviour and its potential as a prognostic biomarker is extensively described in various types of cancer such as: 1) in breast cancer (BC), HOTAIR expression can be induced by estrogen, promoting cell proliferation (Bhan et al., 2014; Abdel-Hamid et al., 2023). Further it acts as a critical modulator of autophagy (Pawłowska, Szczepanska and Blasiak, 2017) and the EMT process (Amicone, Marchetti and Cicchini, 2023); 2) in prostate cancer (PCa), overexpression of HOTAIR can inhibit androgen receptor (AR) degradation, thereby promoting cell growth and invasion (Zhang et al., 2015); 3) in non-small cell lung cancer (NSCLC) cells, HOTAIR regulates proliferation, migration, invasion through the miR-217/DACH1 signalling pathway (S.-S. Chen T. et al., 2019); 4) in colorectal cancer (CRC), HOTAIR upregulation is associated with distant metastasis and a worse prognosis (Tatangelo et al., 2018) by regulating miR-34a expression (Peng et al., 2019); 5) in gastric cancer (GC), HOTAIR promotes migration, invasion, proliferation, and reduced apoptosis via modulating cellular and exosomal miRNAs level (Jie Zhang et al., 2020a); 6) in ovarian cancer, the upregulation of HOTAIR promotes the upregulation of cyclin E, Bcl-2, caspase-3, caspase-9, and several matrix metalloproteinases, thereby modulating cellular proliferation, migration, invasion related with tumour stage, metastases as well as patient survival (Qiu et al., 2015; Yang et al., 2020; Fan et al., 2022); 7) in cervical cancer, endometrial carcinoma and bladder cancer, aberrant HOTAIR expression induces migration and invasion and strongly correlates with poor prognosis (He et al., 2014; Huang et al., 2014; Sun et al., 2015); 8) in oral cancer cells, HOTAIR overexpression increases the tumour progression by sponging miR-326 (Tao et al., 2020), thereby regulating the repression of metastasis-associated gene 2 (MTA2), representing a negative prognostic factor in patients (Zheng et al., 2017); 9) in pancreatic cancer, HOTAIR deregulation promotes stem cells proprieties and induces metastatic switch (Cai et al., 2017; Deng et al., 2021); 10) in melanoma cells HOTAIR represents a marker of metastatic progression also by acting as a sponge for miR-152-3p (Luan et al., 2017).

More recently, circulating HOTAIR can be considered a non-invasive biomarker due to its ability to predict the pathological features of cancer patients (Table 1). In BC, circulating HOTAIR can predict early relapse (Abdel-Hamid et al., 2023) and therapy response (Lu et al., 2018). In GC, HOTAIR expression in serum exosomes is significantly increased and related to metastatic progression (P. Chen L. et al., 2023). In liver cancer (LC), circulating HOTAIR is a potential biomarker used for hepatocellular carcinoma (HCC) detection in cirrhotic livers and prediction of tumour stage (El-Shendidi, Ghazala and Hassouna, 2022). In glioblastoma, melanoma and lung cancer, its circulating levels can predict advanced disease and patient survival (Li et al., 2017; Shen et al., 2018). Furthermore, HOTAIR detection in urine is associated with high-grade muscle-invasive disease in bladder cancer patients (Berrondo et al., 2016) and salivary HOTAIR levels are significantly increased in pancreatic cancer patients compared with healthy controls (Xie et al., 2016).

### The role of HOTAIR in resistance to radiotherapy

The use of radiotherapy often represents the best choice for treating many localized solid tumours, whereas in advanced cancer, radiotherapy, is sometimes used together with chemotherapy, before, during or after the surgical approach (Baskar et al., 2012).

The irradiated cancer cells may develop radioresistance through different mechanisms such as: 1) activation of DNA repair mechanisms (Blaisdell, Harrison and Wallace, 2001; Borrego-Soto, Ortiz-López and Rojas-Martínez, 2015); 2) regulation of the cell cycle and apoptosis (Wu et al., 2023); 3) modulation of EMT and induction of CSCs phenotype (Yu et al., 2019); 4) promotion of tumour cell hypoxia (Beckers, Pruschy and Vetrugno, 2024); 5) influencing epigenetic modulators, especially miRNAs/lncRNAs networks (Cabrera-Licona et al., 2021).

**FIGURE 1:**
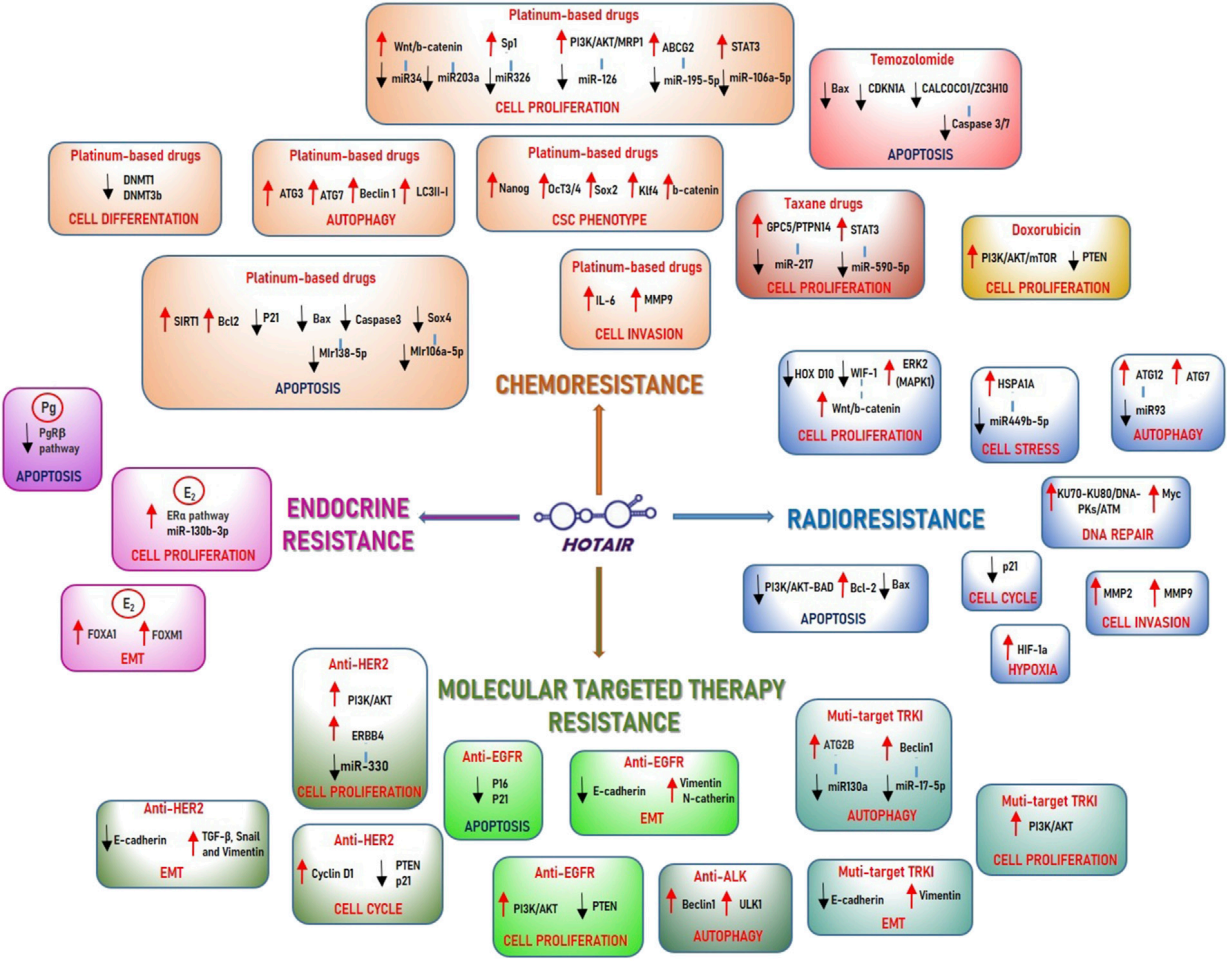
Schematic representation of the HOTAIR role in the anticancer drugs modulation. a) Radiotherapy resistance. HOTAIR can induce radiotherapy resistance by promoting cell proliferation through: 1) deregulation of HOXD10, in breast cancer cells; 2) downregulation of WF-1, with the consequent activation of Wnt/b-catenin pathway, in pancreatic cancer; 3) upregulation of ERK2 pathway in liver cancer cells. HOTAIR can promote DNA damage repair by upregulation of different molecules involved in DNA double-strand break response, such as KU70/KU80, ATM, and by activation of Myc expression in breast cancer cells. HOTAIR is capable of sequestering miR-449b-5p, responsible for HSPA1A repression, thereby upregulating it and modulating cell stress in breast cancer cells. In cervical cancer cells, HOTAIR can promote S phase of the cell cycle by inhibition of p21, and it can induce HIF-1a expression modulating hypoxia. In colon cancer cells HOTAIR can upregulate Bcl2 and downregulate BAX protein, modulating apoptosis, and inducing MMP2 and MMP9 expression promoting cell invasion. HOTAIR can promote autophagy acting as a sponge for miR213, upregulating its target ATG12 in colon cancer cells, and promoting ATG7 expression in pancreatic cancer cells. b) Chemotherapy resistance. HOTAIR is involved in platinum-based drug resistance by modulation of cell proliferation through its interaction with different miRNAs: 1) HOTAIR can sequester miR34 in gastric cancer cells and miR203a in colon cancer cells thereby activating Wnt/b-catenin pathway; 2) in gastric cancer cells HOTAIR acts as a sponge for miR-126, responsible of inhibition of PI3K/AKT pathway and ABC protein, MRP1, and miR195-5p activating ABCG2 protein; 3) HOTAIR can sequester miR-106a-5p inducing STAT3 expression and miR326 to promote the transcription factor, Sp1, respectively in osteosarcoma cells and lung adenocarcinoma cells. HOTAIR is involved in platinum-based drugs resistance by modulation of apoptosis through: 1) the upregulation of the histone deacetylase SIRT1 and Bcl2 in ovarian cancer cells; 2) the downregulation of p21 in leukemia cells; 3) the de-regulation of Bax and caspase3, acting as a sponge for miR138-5p, in ovarian cancer cells; 4) the inhibition of the transcription factor Sox4, acting as a sponge for miR106a-5p, in nasopharyngeal carcinoma cells. HOTAIR is able to contribute to platinum-based drug resistance by modulatiing cell differentiation, through the deregulation of DNA methyltransferase proteins DNMT1 and DNMT3b in small-cell lung cancer. Additionally, it promotes cell invasion by upregulating IL6 and MMP9 in ovarian cancer cells. Moreover, HOTAIR can modulate autophagy, by increase of autophagy-related genes ATG3, ATG7, Beclin1, and LCIII-I in oral squamous cell carcinoma cells, and induce cancer stem cell phenotype, through the upregulation of β-catenin, Nanog, Oct3/4, Sox2 and Klf4 in lung cancer cells. HOTAIR can modulate taxane drug resistance by inducing cell proliferation through the promotion of the metastasis suppressor gene GPC5 and PTPN14, following the sequester of miR217 in gastric cancer cells. Additionally, it upregulates STAT3 after the inhibition of miR590-5p in prostate cancer cells. In doxorubicin resistance, HOTAIR promotes cell proliferation by activation of PI3K/AKT/mTOR pathway in breast cancer cells while it can inhibit apoptosis in temozolomide-resistant cells through deregulation of BAX and CDKN1A, in glioblastoma cells, and CALCOCO1 and ZC3H10 in glioma cells. c) Hormone resistance. In breast cancer cells, HOTAIR is able to induce resistance to endocrine therapies by upregulation of the ERα pathway, thereby promoting cell proliferation, and by the increase of FOXA1 and FOXM1 expression, thereby modulating EMT. HOTAIR promotes progesterone resistance by inhibiting apoptosis through suppression of PRβ in endometrial cancer cells. d) Target therapy resistance. In anti-HER2 drug resistance HOTAIR can promote: 1) cell proliferation, by upregulating ERBB4, after the seizure of its inhibitor miR-330, thereby inducing PI3K/AKT pathway in gastric cancer cells; 2) cell cycle progression by upregulation of Cyclin D1 and deregulation of PTEN and p21 proteins in breast cancer cells; 3) EMT by inducing E-cadherin expression and the reduction of TGF-b, Snail and Vimentin in breast cancer cells. In anti-EGFR therapy, HOTAIR is able to induce resistance by modulation of apoptosis, with the downregulation of p16 and p 21, cell proliferation, by promoting PI3K/AKT pathway, and EMT, by upregulation of E-cadherin and deregulation of N-cadherin and vimentin, in lung cancer cells. HOTAIR contributes to multitarget TKI drug resistance modulating autophagy:1) with the promotion of Beclin1 and ULK1 expression in lung cancer cells; 2) with upregulation of Beclin 1, after the seizure of its inhibitor miR-17-5p, in renal cancer cells; 3) with the induction of ATG2B expression, by sequestering of its inhibitor miR130a, in GIST cells. In TKI drug resistance HOTAIR can also promote EMT by increasing E-cadherin and decreasing Vimentin expression in liver cancer cells and cell proliferation through the induction of PI3K/AKT pathway in leukemia cells.

Numerous studies have highlighted the ability of HOTAIR to modulate sensitivity and resistance to ionizing radiation (Figure 1), especially in BC cell models. Zhou et al. used the MDA-MB231 BC tumour cell line and one non-tumour cell line (MCF-10A) as a control. MDA-MB231 cells are transfected with recombinant plasmid vectors containing the HOTAIR gene. Both cell lines are treated with radiation to evaluate cell growth, proliferation, apoptosis, and DNA breaks. Aberrant expression of HOTAIR can increase radioresistance in MDA-MB231 cells and promotes cell proliferation by modulating HOXD10 expression and the Phosphoinositide 3-kinase/RAC(Rho family) alpha serine-threonine-protein kinase/BCL2 associated agonist of cell death (PI3K/AKT/BAD) pathway (Zhou et al., 2017). Recently these data have been validated *in vivo* in another study. BC cells and mice models are treated with γ irradiation. The upregulation of HOTAIR expression after 4 Gy radiation is strongly associated with the survival rate of BC cells, while HOTAIR silencing significantly decreased the survival rate. In the xenograft model with HOTAIR overexpression a large tumour mass is still clearly visible after 4 Gy radiation (Qian et al., 2020). HOTAIR upregulation promoted DNA damage repair factors KU heterodimer 70 and 80 kDa (KU70/KU80), DNA-PKs, and serine/threonine kinase (ATM) expression. In addition, the mechanism of radioresistance can be associated with the ability of overexpressed HOTAIR to facilitate the recruitment of Enhancer of zeste 2 polycomb repressive complex 2 subunit (EZH2) to the Avian Myelocytomatosis Viral Oncogene Homolog (MYC) promoter (Qian et al., 2020).

Epigenetic mechanisms related to drug resistance often involve the reciprocal interaction of lncRNAs and miRNAs. After radiation therapy, HOTAIR silencing reduced cell proliferation in addition to survival and promoted apoptosis by inducing BAX as well as caspase 3. Furthermore, these cells showed overexpression of miR-218 and were more susceptible to radiation-induced DNA damage. miR-218 blocks decreased apoptosis in HOTAIR knockdown cells after irradiation, suggesting that HOTAIR-miR-218 functional interaction might be involved in the mechanism of radiosensitization (Hu et al., 2019). HOTAIR can also interact with miR-449b-5p to modulate radioresistance in BC cells. Zhang et al. demonstrated that the expression level of HOTAIR is inversely correlated with the radiosensitivity of cells and directly related to heat shock protein A (Hsp70) family member 1A (HSPA1A) in BC tissues. HOTAIR induced HSPA1A expression in irradiated cells, acting as a sponge for miR-449b-5p and thereby relieving the HSPA1A repression mediated by miR-449b-5p (Shuqin Zhang et al., 2020d).

HOTAIR also induced radio-resistance in cervical cancer. Jing et al. showed that HOTAIR upregulation can predict the radioresistance of cervical cancer patients. HOTAIR silencing can modulate the sensitivity to radiotherapy in HeLa cells. The inhibition of p21 can alter this process. Knockdown of HOTAIR-induced cell cycle arrest in the G1 phase in these cells, which is voided by p21 silencing. HOTAIR overexpression in parental cells, C33A cells, significantly inhibited the p21 protein level and promoted the S phase of the cell cycle. In the same way, the knockdown of HOTAIR suppressed tumour growth *in vivo* and further increased the radiosensitivity of cervical cancer. In tumour samples, immunohistochemistry (IHC) results showed the downregulation of Ki-67 and the upregulation of p21 (Jing et al., 2015). The relation between HOTAIR and hypoxia in the modulation of radioresistance has been also described. Li et al. analysed the expression of Hypoxia-Inducible Factor-1alpha (HIF-1α) and HOTAIR in cervical cancer cells after radiation. Aberrant expression of HOTAIR strongly induced HIF-1α expression in cervical cancer cells and in mice models exposed to radiation. Silencing of HIF-1α can increase cell invasion and decrease apoptosis, induced by upregulation of HOTAIR, in cervical cells (Li et al., 2018).

In CRC, HOTAIR silencing inhibited CCL244 cell proliferation by inducing the cell cycle arrest in the G0/G1 phase, thereby leading radiosensitization in a dose-dependent manner in CRC cells. The role of HOTAIR in radiation-induced apoptosis has been abundantly discussed. Yang et al. showed that HOTAIR knockdown in irradiated cells leads to the deregulation of anti-apoptotic protein Bcl-2 and the induction of pro-apoptotic protein BAX expression. Furthermore, the knockdown of HOTAIR in irradiated CRC cells led to a decreased expression of metalloproteinase 2 (MMP2) and metalloproteinase 9 (MMP9) suggesting a role of HOTAIR in influencing the invasion and migration of colon cancer cells after irradiation (Yang et al., 2016). Recently, Huang et al. highlighted that HOTAIR can be detected in the plasma of CRC patients after radiotherapy treatment and *in vitro* in CRC cells after ionizing radiation administration (Liu et al., 2020). HOTAIR silencing is capable of reducing cell survival fractions and cell viability, increasing cell apoptosis, and reducing IR-induced autophagy in irradiated SW480 and HCT116 CRC cells. This may suggest that HOTAIR silencing can increase the radiosensitivity of CRC cells by the inhibition of autophagy. To study the mechanism involving HOTAIR in CRC radioresistance, a bioinformatics analysis revealed a strong relation between HOTAIR and miR-93. Additionally, it identified Autophagy Related 12 (ATG12), a part of autophagy molecular complexes, as a potential target of miR-93. Huang et al. described that HOTAIR induced ATG12 expression in CRC cells, acting as a molecular sponge for miR-93. In addition, in CRC xenograft tumours, HOTAIR silencing increased the radiosensitivity through the modulation of autophagy mediated by miR-93/ATG12 axis (Liu et al., 2020).

HOTAIR is also involved in the modulation of radioresistance in pancreatic cancer. HOTAIR expression is overexpressed in the pancreatic ductal adenocarcinoma (PDAC) cell lines and tissues and its silencing enhances the radiosensitivity of PDAC cells, reduces the proliferation, and increases the apoptosis of irradiated cells (Jiang et al., 2016). Jiang et al. suggested that WIF-1 interaction with HOTAIR can influence the radiosensitivity of PDAC cells (Jiang et al., 2016). In addition, HOTAIR silencing increased the expression of WNT Inhibitory Factor 1 (WIF-1), a primary inhibitor of the Wnt/β-catenin pathway. It has been demonstrated that the suppression of the Wnt/β-catenin signalling pathway increased the radiosensitivity in other cancers (Wang et al., 2014). Radiation treatment can induce aberrant expression of HOTAIR in PDAC cells in time- and dose-dependent manners and HOTAIR silencing can enhance the radiosensitivity of these cells. HOTAIR is also able to increase the number of autophagosomes promoting autophagy in these cells. Mechanistically, HOTAIR promoted autophagy by targeting the ATG7. ATG7 expression is significantly induced by HOTAIR both at mRNA and protein levels. On the contrary, the knockdown of HOTAIR decreased ATG7 expression (Wu et al., 2018).

Pei et al. revealed that HOTAIR is upregulated in LC tissues and LC stem cells (LCSCs), and correlated to stemness maintenance and radioresistance (Pei et al., 2023). HOTAIR knockdown decreased the stemness of LCSC cells, while its upregulation reversed this effect, as demonstrated by microsphere formation assay data. Mechanistically, Pei et al. highlighted that the transcription repressor LSD1 could interact with HOTAIR, preserving the stemness of LCSC cells and decreasing their sensitivity to radiotherapy treatment. HOTAIR may mediate the binding of LSD1 to the mitogen-activated protein kinase 1 (MAPK1 or ERK2) promoter. In this way, HOTAIR can promote the demethylation of H3K9me2 on the ERK2 promoter, regulating its expression. Silencing of LSD1 attenuated the ability of LCSCs to form microspheres. X-ray irradiation of LCSC cells and LSD1 knockdown decreased cell proliferation, while ERK2 upregulation reversed this condition. These data suggested that the mechanism of radioresistance is modulated by LSD1/ERK2 axis to preserve the stemness of LCSCs. Furthermore, the expression of HOTAIR is regulated by the epigenetic modulator Jumonji domain-containing 6 (JMJD6)– bromodomain containing 4 (BRD4) complex, by binding to its promoter region. JMJD6 silencing can induce radiosensitivity of LC cells by HOTAIR and ERK2 inhibition suggesting that the JMJD6-BRD4 complex and HOTAIR-LSD1-ERK2 axis play a crucial role in the irradiation tolerance of LCSCs (Pei et al., 2023).

### The role of HOTAIR in chemotherapy resistance

Chemotherapy is crucial in managing tumour growth and extending patients’ survival rates. Despite the emergence of targeted therapies as a promising avenue in cancer treatment, chemotherapy remains the prevalent choice, despite its well-documented adverse effects on both physical and mental wellbeing (Anand et al., 2023). Further acquired resistance is a great challenge for chemotherapy and is strongly related to tumour recurrence and an increase in mortality. Recent studies revealed that HOTAIR overexpression plays a crucial role in tumour initiation and progression, and is an important modulator of chemoresistance (Figure 1).

Guo et al. verified the high expression of HOTAIR in NSCLC samples of patients receiving neoadjuvant chemotherapy compared to cancer-adjacent tissue samples, correlating the high expression of this lncRNA with poor prognosis. Moreover, the silencing of HOTAIR (si-HOTAIR) in NSCLC-resistant cell lines to cisplatin increased the sensitivity to chemotherapy by reducing the protein expression levels of wingless integrated 3a (Wnt3a), β-catenin and Adenomatous Polyposis Coli (APC), key regulators of the Wnt signalling pathway (Guo et al., 2018). Liu et al. obtained similar results, showing an elevated HOTAIR expression in tissues of NSLCS patients with cisplatin resistance compared to samples with non-cisplatin resistance. These data, supported by *in vitro* results, indicated that elevated HOTAIR expression is implicated in cisplatin resistance. Interestingly, the authors identified a correlation between the HOTAIR expression and tumour stem cell formation. In particular, the *in vitro* results showed the ability of A549 cisplatin-resistant cells to form spheres is greater than that of A549 cells, modulating the expression levels of tumour stem cell biomarkers, such as β-catenin, Nanog, octamer-binding transcription factor 3,4 (October 3, 4), SRY-box transcription factor 2 (Sox2), c-Myc and KLF transcription factor 4 (Klf4) (Liu et al., 2016). Li et al. showed the role of HOTAIR in cisplatin resistance by modulating the expression of miR-326 and Sp1. More precisely, si-HOTAIR resulted in an increase in miR-326 expression, downregulation of Sp1 expression, and the subsequent restoration of sensitivity to cisplatin in lung adenocarcinoma (LAD) models, in both *in vitro* and *in vivo* lung adenocarcinoma (LAD) models (Li et al., 2016). Silencing of HOTAIR resulted in increased drug sensitivity, enhanced apoptosis, and slowed down the progression of the cell cycle in both *in vitro* small cell lung cancer (SCLC) cell lines and *in vivo* models. Moreover, the authors also validated that HOTAIR mediated the chemoresistance in SCLC cells by regulating HOXA1 methylation through decreasing DNA-methyltransferase 1 (DNMT1) and DNMT3b expression (Fang et al., 2016).

In ovarian cancer, Wang et al. showed that the HOTAIR expression level is higher in late-stage malignant ovarian tissue tumours *in vitro* cell models resistant to cisplatin treatments compared to the level in early-stage tumours. The same data are confirmed in vitro cell models resistant to cisplatin treatments. The si-HOTAIR triggered inhibition of cell proliferation, reduction of cell invasion ability and notably restored cisplatin sensitivity in SKOV-3CDDP/R cisplatin-resistant epithelial ovarian cancer cell line by enhancing cytotoxicity and apoptosis rate (Wang et al., 2015). In another compelling study, the authors proved that HOTAIR knockdown led to the upregulation of miR-138-5p in SKOV3/DDP and A2780/DDP cisplatin-resistant cell lines. Mechanically, HOTAIR silencing restored cisplatin sensitivity by increasing miR138-5p expression level, decreasing expression of EZH2, SIRT1, and Bcl2, and markedly increasing protein levels of BAX, CI-caspase 3 and CI- Poli ADP-ribose polymerase (PARP) (Yun Zhang et al., 2020e). Yu et al. showed that silencing of HOTAIR can reverse the resistance of ovarian cancer cells to cisplatin by disrupting the autophagy process triggered by the drug. In particular, downregulating HOTAIR decreased the levels of autophagy-related proteins such as ATGF7 and LC3II-I, resulting in a reduction in the expression of the anti-apoptotic protein Bcl-2 as well as an elevation in the levels of the pro-apoptotic protein BAX (Yu et al., 2018). In another study, HOTAIR deregulation restored the sensitivity to chemotherapy in vitro and *in vivo* models. HOTAIR suppression led to cell cycle arrest, in the G2/M phase, by regulating the expression of CHECK1. This checkpoint kinase plays an essential role in cell cycle regulation and DNA damage response (Jiang et al., 2020). Conversely, Ozes et al. investigated the impact of HOTAIR on DNA methylation and damage. These findings indicated that HOTAIR plays a crucial role in both cellular senescence and sensitivity to platinum-based treatments. Specifically, the ectopic expression of HOTAIR-induced Nuclear factor kappa B (NF-kB) activation during DNA damage response and increased expression of MMP9 and Interleukin-6 (IL-6), both key NF-kB target genes. Moreover, these research findings highlighted that the NF-kB-HOTAIR pathway is involved in DNA damage processes, leading to cellular senescence and contributing to chemoresistance in epithelial ovarian cells (Özeş et al., 2016). In BC, Li et al. confirmed the role of HOTAIR in doxorubicin resistance. Indeed, HOTAIR silencing can reduce cell proliferation, induce the apoptosis rate, and impede the activation of PI3K/AKT/mTOR signalling in doxorubicin-resistant BC cell lines (Li et al., 2019). Tang et al. compared the expression levels of exosomal HOTAIR from the serum of BC patients, tumour tissues, and cell culture medium. Intriguingly, the obtained data showed that higher levels of exosomal HOTAIR are associated with a decreased response to neoadjuvant chemotherapy and tamoxifen hormone therapy in BC patients (Tang et al., 2019). In GC, Yan et al. described that HOTAIR expression is significantly increased in cisplatin-resistant gastric cells and 30 pairs of GC tissue specimens. Using gain and loss of function approaches, the authors showed that the overexpression of HOTAIR promoted GC cell proliferation, caused cell cycle arrest in G1/S phase, and reduced apoptosis; while silencing HOTAIR increased sensitivity to cisplatin treatment in GC cells. Additionally, high expression of HOTAIR acted as a competitive endogenous RNA (ceRNA) by regulating the expression of miR-126. This leads to a negative correlation between miR-126 and HOTAIR expression in both GC lines and human tissues. The inhibition of miR-126 expression induced the expression of miR-126 target genes, such as Vascular endothelial growth factor (VEGFA), PI3KR2, PI3K, AKT, and Multidrug resistance-associated protein 1 (MRP1), which are involved in the PI3K/AKT/MRP1 oncogenic signalling pathway (Yan et al., 2016). Cheng et al. validated the role of HOTAIR in regulating cisplatin resistance in GC. They also established that HOTAIR is upregulated in GC tissues and SGC7901, SGC7901/DDP, MGC803, and MGC803/DDP cell lines compared to health control samples. Its expression level is inversely related to the expression of miR-34a. Mechanistically, the knockdown of HOTAIR restored cisplatin sensitivity in both *in vitro* and *in vivo* experiments by blocking the PI3K/AKT and Wnt/β-catenin signalling pathways via miR-34a (Cheng et al., 2018). Luo and colleagues recently investigated how HOTAIR contributes to the progression and resistance to oxaliplatin in GC. The data obtained highlighted the relation between HOTAIR and ABCG2, a member of the ABC transporter superfamily, via miR-195-5p. HOTAIR acted as a sponge for miR-195-5p, facilitating ABCG2 expression and consequently promoting the proliferation of GC cells, thereby leading to oxaliplatin resistance (Luo et al., 2023). The atypical expression of HOTAIR has been primarily observed in advanced stages (III and IV) of GC, and has been linked to paclitaxel and doxorubicin resistance. The authors highlighted an inverse correlation between HOTAIR and miR-217 in GC tissues. Mechanistically, HOTAIR suppressed miR-217 expression and increased the expression of glypican-5 (GPC5) and Protein Tyrosine Phosphatase Non-Receptor Type 14 (PTPN14) promoting paclitaxel and doxorubicin resistance in GC cells (H. Wang et al., 2018a).

A series of studies have confirmed that HOTAIR played a significant role in CRC by affecting the effectiveness of platinum-based drugs. Xiao et al. exhibited a crosstalk between HOTAIR and miR203a-3p, in both clinical tissue samples and *in vitro* cell lines. Remarkably, HOTAIR depletion and miR-203a-3p overexpression in CRC cells arrested cell growth and partially restored chemosensitivity by blocking the Wnt/β-Catenin signaling pathway (Xiao et al., 2018). Separate studies tried to identify novel lncRNAs as predictive markers of chemoresistance using integrative bioinformatics analysis. In particular, Sun et al. identified four hubs of lncRNAs that played a crucial role in the mechanisms of action of oxaliplatin and irinotecan resistance, including Colorectal Neoplasia Differentially Expressed (CRNDE), H19, Urothelial Cancer Associated 1 UCA1) and HOTAIR. Among these, HOTAIR overexpression is linked to advanced tumour node, metastases, drug response, and a worsened CRC prognosis (Sun, Liang and Qian, 2019). Wei et al. constructed a ceRNA network, based on RNA information obtained from bioinformatics analyses, to identify new players in oxaliplatin resistance. Specifically, the ceRNA network developed by the authors contained 503 lncRNA-miRNA-mRNA regulatory, 210 lncRNA-miRNA pairs, 382 miRNA-mRNA, and 212 mRNA co-expression pairs. Among these, HOTAIR and 14 mRNAs significantly correlated with patient prognosis for oxaliplatin resistance (Wei et al., 2019).

In oral squamous cell carcinoma (OSCC), Wang et al. described that HOTAIR suppression reduced the formation of autophagosomes by downregulating the expression of autophagy-related genes such as beclin1, ATG3, ATG7, and MAP1LC3B. Additionally, si-HOTAIR enhanced the rate of apoptosis and increased the sensitivity to cisplatin in KB and CAL-27 OSCC cells (X. Wang et al., 2018b). The aberrant expression of HOTAIR, together with upregulation of EZH2, has been detected in laryngeal squamous cell carcinoma (LSCC) in which it is significantly associated with T-phase, pathological grades, and the risk of lymphatic metastases. The role of HOTAIR in cisplatin resistance by modulation of EMT has been demonstrated in both LSCC *in vitro* and *in vivo* models. The crosstalk between HOTAIR and miR-613, regulating Snail Family Transcriptional Repressor 2 (SNAI2) expression, could modulate this process (Zhou et al., 2018). HOTAIR is overexpressed in both cisplatin-resistant tissues and cell lines of nasopharyngeal carcinoma (NPC) and its silencing increased sensitivity to cisplatin in DDP-resistant NPC cells, suppressed cell viability, invasion, and migration, and promoted apoptosis by modulating the activity of the miR-106a-5p/SOX4 axis (Cao et al., 2022). The crucial role of HOTAIR in the induction of temozolomide (TMZ) resistance in glioblastoma cells has been also described. In glioblastoma multiforme (GBM), Zang et al. reported that the expression of HOTAIR is positively correlated with the expression of Hexokinase 2 (HK2), an essential enzyme in the glycolytic pathway involved in aerobic glycolysis. The HOTAIR knockdown suppressed the HZ2 expression, inhibited cell proliferation, and enhanced the cytotoxicity of TZM in both U87 and A172 cell lines and in vivo mice models. Mechanistically, the authors illustrated that HOTAIR acted as the upstream mediator of HK2 by sequestering miR-125, thereby impairing the glycolysis balance in GBM (Jinnan Zhang et al., 2020b). Furthermore, Zhao et al. identified a novel small-molecule inhibitor, EPIC-0412, capable of reversing TMZ resistance *in vitro* and *in vivo* experiments. The authors found interesting, that the inhibitor EPIC-0412 physically disrupts the interaction between HOTAIR and EZH2, resulting in increased levels of cyclin-dependent kinase inhibitor 1A (CDKN1A) and BAX, ultimately inducing cell cycle arrest in addition to apoptosis in GBM cells. Moreover, EPIC-0412 improves the therapeutic efficacy of TMZ by inhibiting the DNA damage repair mechanism via epigenetic pathways (Zhao et al., 2023). Wang et al. revealed that HOTAIR is delivered by extracellular vesicles (EVs) found in the serum of patients. The effects of HOTAIR delivered by serum-EVs increased aggressive phenotype, tumour growth, and TMZ resistance in both *in vitro* and *in vivo* experiments. Serum-EVs suppressed miR-526b-3p-mediated inhibition of epithelial V-like antigen 1 (EVA1), which has been verified to be implicated in tumour progression and drug response (Wang et al., 2022). HOTAIR can also repress two crucial target genes, Calcium Binding And Coiled-Coil Domain 1 (CALCOCO1) and zinc finger CCCH-type containing 10 (ZC3H10), directly involved in the modulation of sensitivity to TMZ in U251 glioma cells (Lei Zhang et al., 2020c).

In many other solid tumours HOTAIR is described as one of the potential modulators of chemoresistance mechanism: 1) in PCa, Wang et al. demonstrated the contribution of HOTAIR to the expansion and growth of prostate cancer stem-like cells (PCSLCs), which are considered one of the mechanisms that contribute to drug resistance. Interestingly, the authors showed that HOTAIR functioned as a miR-590-5p sponge, leading to the upregulation of IL-10 and subsequent activation of the Signal transducer and activator of transcription 3 (STAT3). This mechanism is capable to induce docetaxel (Doc) resistance in both C4-2 PCa cells and *in vivo* animal models (N. Wang et al., 2020); 2) in bladder transitional cell carcinoma tissue (TCC), as well as in T24 and J82 cell lines, overexpression of HOTAIR led to an increase in cell proliferation, inhibition of chemosensitivity to doxorubicin, in addition to a reduction in the rate of apoptosis. Silencing HOTAIR reverted these processes (Shang et al., 2016); 3) in cervical cancer, HOTAIR is involved not only in the promotion of proliferation and migration but also in the induction of EMT and cisplatin, paclitaxel, as well as docetaxel resistance. HOTAIR may contribute to the malignant phenotype and chemoresistance through competitive binding with miR-29b and by the indirect regulation of PTEN via SP1, followed by the inhibitory regulation of PTEN on PI3K (Zhang et al., 2022); 4) in osteosarcoma (OS), HOTAIR and STAT3 are significantly increased, while miR-106a-5p is dramatically decreased in cisplatin-resistant (DDP) OS tissues as well as Saos/DDP, MG-63/DDP, and U2OS7DDP cells. Mechanistic studies confirmed that HOTAIR affects cell proliferation, invasion, migration, and apoptosis through the miR-106a-5p/STAT3 axis (Guo et al., 2020); 5) in HCC, HOTAIR overexpression and the downregulation of miR-34a is identified in taxol-resistant HepG2 and SMMC7721 cells. HOTAIR knockdown inhibited cell growth and invasion while enhancing cell death by increasing the expression of miR-34a, which in turn led to the activation of the AKT and Wnt/β-catenin signalling pathways in Taxol-resistance HCC cell lines (Duan et al., 2020); 6) in pancreatic cancer, Wang et al. suggested that HOTAIR overexpression is inducted by gemcitabine treatment in pancreatic CSCs triggering acquired resistance. The ectopic expression of HOTAIR resulted in increased cell proliferation and migration as well as in the maintenance of the self-renewal capability of pancreatic CSCs (L. Wang et al., 2017b).

Finally, HOTAIR’s role as a chemoresistance biomarker has also been demonstrated in several haematological tumours. In acute myeloid leukemia (AML), Ling-Li and colleagues discovered that the expression of HOTAIR is elevated in drug-resistant K562/AO2 leukemia cell lines and bone marrow samples from patients with refractory and relapsed AML. Inhibition of HOTAIR suppressed proliferation, increased the rate of apoptosis, and enhanced sensitivity to chemotherapy by regulating the expression of the P21 and AKT/Notch1 signalling pathways (M.-L. Li D. et al., 2020). In addition, Zhou et al. showed a higher expression of HOTAIR and a lower PTEN expression in samples of patients with relapsed and refractory AML and doxorubicin-resistant cell lines compared to control samples. Interestingly, the results showed that HOTAIR suppressed PTEN expression levels by upregulating DNMT3b-dependent way and this mechanism conferred doxorubicin resistance in AML (Zhou et al., 2021). Moreover, Liu et al. investigated the role of Curcumin, an active flavonoid component of Curcuma longa herb, in the resistance of ALM to adriamycin. They demonstrated that Curcumin inhibited cell proliferation and migration, plus hampered cell cycle progression and made HL-60/ADR cell lines more responsive to Adriamycin by controlling HOTAIR/miR-20a-5p/WT1 axis (Liu J. et al., 2021). Additionally, HOTAIR played a significant role in dexamethasone (DEX) chemoresistance of multiple myeloma (MM) cells. HOTAIR silencing suppressed MM cell viability, arrested the cell cycle at the G0/G1 phase, and increased sensitivity to DEX by regulating the Janus Kinase 2 (JAK)/STAT3 signalling pathway (Guan et al., 2019).

### The role of HOTAIR in endocrine drug resistance

Endocrine therapy works by preventing the functions of estrogen and/or progesteron at the receptor level or by reducing their production. Various types of endocrine therapies have been developed, such as selective estrogen receptor (ER) modulators, selective estrogen receptor downregulators, aromatase inhibitors (AIs), and luteinizing hormone-releasing agonists (Lumachi et al., 2011).

A major challenge in treating ER-positive BC is to overcome endocrine resistance (García-Becerra et al., 2012). Mechanisms of endocrine resistance include deregulation of ER expression, mainly due to acquired mutations in the gene (Merenbakh-Lamin et al., 2013; Robinson et al., 2013; Toy et al., 2013; Jeselsohn et al., 2014), epigenetic alterations of ER (Achinger-Kawecka et al., 2020), post-translational modification, modifications of cell cycle regulators, and deregulation of cellular responses of endocrine agents (Murphy and Dickler, 2016). Among epigenetic modifications, a main role in endocrine resistance has been described for ncRNAs, especially lncRNAs (Horie et al., 2022; Yang et al., 2022; Quttina et al., 2023; Yu et al., 2023).

The function of HOTAIR has been well documented in breast tumours, in which it is upregulated in metastatic BC (Gupta et al., 2010) and associated with different mechanisms of resistance to anticancer therapies (Figure 1).

Xue et al. showed that HOTAIR expression level is significantly higher in tamoxifen-resistant BC than in primary, hormone-naïve tumours. To analyse the role of HOTAIR in tamoxifen resistance, they generated a tamoxifen-resistant (TamR) MCF-7 cell line in which the level of HOTAIR increased 4-fold following long-term treatment with tamoxifen. HOTAIR silencing significantly reduced the growth of tamoxifen-resistant MCF7 cells. Furthermore, clonogenic assays highlighted that the knockdown of HOTAIR significantly inhibited the colony-forming ability of tamoxifen-resistant cells, further supporting the idea that HOTAIR played an important role in promoting the growth of tamoxifen-resistant BC cells (Xue et al., 2016). Milevskiy et al. showed that HOTAIR expression is downregulated by estrogen and upregulated by Forkhead-box (FOX) A1 and FOXM1 in BC. HOTAIR alone can stratify the survival of patients who received chemotherapy in combination with endocrine therapy, while FOXM1 alone is a significant biomarker for response to tamoxifen. Combined expression of HOTAIR and FOXM1 increased the predictive value of these biomarkers for all endocrine therapies alone or in combination with chemotherapy (Milevskiy et al., 2016). HOTAIR expression is detected *in vitro* models of estrogen deprivation or resistance to MCF7-derived antiestrogen cell lines, MCF7X, TAMR, and FASR. The expression levels of HOTAIR are increased, and the expression of ER1, FOXM1, and FOXA1 is altered in MCF7X and TAMR (Milevskiy et al., 2016). A recent bioinformatic analysis was performed to analyse differentially expressed genes (DEGs), differentially expressed miRNAs (DEMs), and differentially expressed lncRNAs (DELs) in long-term estrogen-deprived (LTED) ER-positive BC cells, to create an mRNA-miRNA-lncRNA regulatory network associated with endocrine resistance. The mRNA-miRNA-lncRNA network included 60 mRNA nodes, 6 miRNA nodes and 3 lncRNA nodes. The hub genes in the network mRNA-miRNA-lncRNA are ABCG2, ER1 and GJA1. Moreover, Zhang et al. suggested the potential role of the ER1/miR-130b-3p/HOTAIR axis in regulating endocrine resistance in BC (M. Zhang et al., 2021b).

Progesterone is a key hormone in the endometrium where it is capable of blocking estrogen-driven cell growth activity. If progesterone levels are low, the uncontrolled activity of estrogen could lead to the development of endometrial hyperplasia and adenocarcinoma (Gompel, 2020). In these cases, treatment with progestins is suggested, but frequently endometrial tumours can become resistant to treatment. The mechanisms of progesterone resistance are poorly understood, but even in this case, epigenetic alterations may be involved (Al-Sabbagh, Lam and Brosens, 2012).

To explore the involvement of HOTAIR in the development of progesterone resistance in endometrial cancer cells, Chi et al. analysed the relationship between HOTAIR and progesterone receptor (PR) Beta expression in endometrial carcinoma (EC) tissues and cell lines (Chi et al., 2019). An inverse correlation between HOTAIR and PRB expression is observed in both EC tissues and cells. The negative correlation might suggest that HOTAIR promotes progesterone resistance through suppressing PRB (Figure 1). Silencing of HOTAIR significantly induces the expression of PRB in EC cells and promotes the sensitivity of cells to medroxyprogesterone acetate (MPA). PRB downregulation reverses this condition. Moreover, when HOTAIR and LSD1 were both downregulated, it resulted in an increase in PRB expression and enhanced apoptosis triggered by MPA. These data suggested that HOTAIR silencing promoted PRB transcription by recruiting LSD1, causing H3K4me2 demethylation at the PRB promoter (Chi et al., 2019).

### The role of HOTAIR in resistance to molecular targeted therapy

Molecular targeted therapeutic cancer drugs included different agents with specific characteristics. They are sorted into categories based on their characteristics, including small molecules, monoclonal antibodies, immunotherapeutic cancer vaccines, and gene therapy agents (Lee, Tan and Oon, 2018). Targeted therapies can act on cell surface antigens, growth factors, receptors, or signal transduction pathways, especially tyrosine kinase molecules, involved in cell cycle progression, cell death, metastasis, and angiogenesis (Liu, Pandya and Afshar, 2021b). Furthermore, these drugs can target cancer cells as well as the constituents within the TME to activate the immune system (Xiao and Yu, 2021).

Tyrosine kinase inhibitors (TKI) are a group of pharmacologic agents, small molecule and antibody-based drugs that disrupt the signal transduction pathways of protein kinases. They are used in the treatment of most solid tumours as well as haematological malignancies and currently, there are over 50 U.S. Food and Drug Administration (FDA)-approved TKIs (Latham, Geffert and Jackson, 2024). The human EGFR family consists of four members (ErbB1–4) (Roskoski, 2019). These receptors have a protein kinase domain targetable by different small molecule and antibody-based drugs. For example, trastuzumab and pertuzumab are monoclonal antibodies that target the extracellular domain and are used for the treatment of ErbB2/HER2-positive tumours (von Minckwitz et al., 2017). Chen and colleagues demonstrated that HOTAIR expression is markedly upregulated in trastuzumab-resistant BC cell lines (Figure 1). EMT is also altered with the upregulation of TGF-β, Snail and Vimentin, in addition to the downregulation of E-cadherin. The silencing of HOTAIR sensitized the cells to trastuzumab leading to the downregulation of TGF-β, Snail, Vimentin, p-AKT, p-APK and CyclinD1 and the upregulation of E-cadherin, PTEN and P27 (T. Chen S.-S. et al., 2019). Likewise, in GC HOTAIR levels are inversely related to sensitivity to trastuzumab and its aberrant expression can promote the proliferation as well as invasion of GC cells (Bie et al., 2020). Bie et al. confirmed these results showing that HOTAIR expression level is negatively correlated with the sensitivity to trastuzumab in primary GC patients and its aberrant expression caused specific trastuzumab resistance in both cells and nude mice models. Mechanistically, HOTAIR can competitively bind miRNA330 through the ceRNA network, resulting in increased expression of ERBB4. Overexpression of ERBB4 in turn can increase phosphorylation of the PI3K/AKT pathway protein, thereby promoting GC cell resistance to trastuzumab (Figure 1) (Bie et al., 2020).

The approved EGFR-TKIs can be divided into first-, second-, third-, and fourth-generation TKIs, however, the information in the literature regarding the role of HOTAIR in anti-EGFR drugs refers only to first-generation drugs (Figure 1). The molecular mechanism by which HOTAIR induced gefitinib resistance in NSCLC is unclear because it appears modulated by different pathways. Li and colleagues also found that HOTAIR expression is increased in gefitinib-resistant lung cancer tissues and that its overexpression can lead to enhanced drug resistance in lung cancer cell models. They showed that HOTAIR aberrant expression promoted cell cycle progression through epigenetic regulation of EZH2/H3K27. Silencing of EZH2 sensitized the lung cancer cells to gefitinib and induced expression of p16 and p21, suggesting that HOTAIR contributes to gefitinib resistance by regulating EZH2/p16/p21 axis (Li et al., 2021).

Among multi-target tyrosine receptor kinase inhibitors (TRKI) resistances, imatinib resistance is one of the most studied mechanisms, and HOTAIR seems to play a significant role in regulating this process. It is known that lncRNA HOTAIR is upregulated in recurrent gastrointestinal tumours (GISTs) (Bure et al., 2018). Zhang and colleagues investigated changes in lncRNA expression following treatment with imatinib, discovering that HOTAIR was markedly deregulated (Figure 1). Interestingly, after imatinib treatments, HOTAIR translocated from the nucleus to the cytoplasm in GIST cells. Its silencing induced suppression of autophagy and imatinib sensitivity in GISTs. In addition, HOTAIR negatively correlated with miRNA-130a, and silencing miRNA-130a can restore drug sensitivity. In addition, authors identified autophagy-related protein 2 homolog B (ATG2B) as a downstream target of miR-130a and HOTAIR. These data are confirmed also *in vivo* mouse tumour models suggesting that HOTAIR targets the ATG2B inhibitor miR-130a to upregulate the level of cell autophagy thereby promoting the imatinib resistance in GISTs (J. Zhang et al., 2021a).

Imatinib is also the drug of choice in the treatment of chronic myelogenous leukemia (CML) but knowledge of resistance mechanisms in this tumour is limited. HOTAIR appears upregulated in imatinib-resistant cells K562. HOTAIR silencing in these cells decreased PI3K/Akt pathway activation, indicating that it may be key to improving acquired resistance to imatinib in CML cells through this signaling pathway (H. Wang et al., 2017a).

Yang et al. highlighted that silencing HOTAIR inhibited resistance to anti-Anaplastic kinase lymphoma (ALK) and ROS Proto-Oncogene 1 (ROS1) inhibitor, Crizotinib, suppressing tumour progression by promoting apoptosis in NSCLC cells. Moreover, HOTAIR suppression inhibited the expression level of the phosphorylated form of Unc-51 Like Autophagy Activating Kinase 1 (ULK1) and Beclin1, suggesting the crucial role of HOTAIR in drug resistance by inhibiting the autophagy process via the ULK1 pathway (Figure 1) (Yang et al., 2018).

Sorafenib and sunitinib are TRKI-approved by the FDA for the treatment of different solid tumours. In particular, sorafenib has been authorized as a unique target cancer drug for the treatment of advanced HCC, but even in this case the mechanisms of resistance are poorly understood (W. Tang et al., 2020a). Tang et al. observed that HOTAIR overexpression is associated with the increase of sorafenib resistance in HCC cells. Its silencing promoted sorafenib sensibility on HCC cells restoring EMT via increasing E-cadherin and decreasing Vimentin expression. The downregulation of HOTAIR also led to the upregulation of miR-217 described as a tumour suppressor in different human cancers (X. Tang et al., 2020b). In another study, Han et al. analysed HOTAIR expression in both tissue and blood of HCC patients showing its upregulation in both biological matrices, with a significant correlation between the two HOTAIR expression levels. Moreover, overexpression of HOTAIR is associated with survival in advanced HCC patients treated with sunitinib, suggesting that the expression level of HOTAIR can be a useful tool in predicting the effectiveness of sunitinib therapy (Figure 1) (Han et al., 2022).

The predictive value of HOTAIR for sunitinib therapies has also been validated in renal cancer (D. Li M.-L. et al., 2020). Sunitinib-resistant cells (786-O-R and ACHN-R) are constructed using parental RC cells (786-O and ACHN). HOTAIR is overexpressed in sunitinib-resistant cells compared to parental cells. Silencing HOTAIR can impact autophagy in renal cancer models in both *in vitro* and *in vivo* models, restoring drug sensitivity in sunitinib-resistant cells. HOTAIR may compete with miR-17-5p to regulate Beclin1 expression. Knockdown of miR-17-5p in parental cells increases the resistance of cells to sunitinib while its overexpression in sunitinib-resistant cells increases the sensitivity of cells to sunitinib. Li et al. demonstrated that miR-17-5p is poorly expressed in sunitinib-resistant cells compared to parental cells and that HOTAIR knockdown is capable of restoring miR-17-5p expressions in resistant cells. These data suggested that the mechanism of sunitinib resistance in renal cancer cells is modulated by the ability of HOTAIR to negatively target miR-17-5p to activate autophagy (Figure 1) (D. Li M.-L. et al., 2020).

### The role of HOTAIR in the modulation of immune escape

The modulation of immune cells in TME is closely linked to immune escape and plays a crucial role also in drug resistance. For this reason, the development of immunotherapies such as immune checkpoint blockade (ICB) and adoptive cell therapy (ACT) has drastically changed the landscape of cancer therapy. Recently, several antibodies targeting cellular immune checkpoints (e.g., Programmed death 1 (PD-1)/Programmed death ligand 1 (PD-L1) and Cytotoxic T-Lymphocyte Antigen 4 (CTLA-4)) have been developed to promote the activation of T cells and subsequent tumour immune escape control (Shiravand et al., 2022). Numerous lncRNAs are expressed in immune cells and play essential roles in controlling immune response because they are involved in T cell activation/differentiation (Guo et al., 2015; Chen, Satpathy and Chang, 2017). They can regulate cytotoxicity CD8^+^ T cells recruitment in the TME, and ultimately promoting tumour progression (Wu et al., 2017). LncRNAs have the ability to promote an immunosuppressive TME by activating various signalling pathways, such as TNF-α/NF-κB, PI3K/AKT, Wnt/-catenin, and JAK/STAT pathways, thereby modulating tumour escape and inducing drug resistance (Mofed et al., 2022).

**FIGURE 2:**
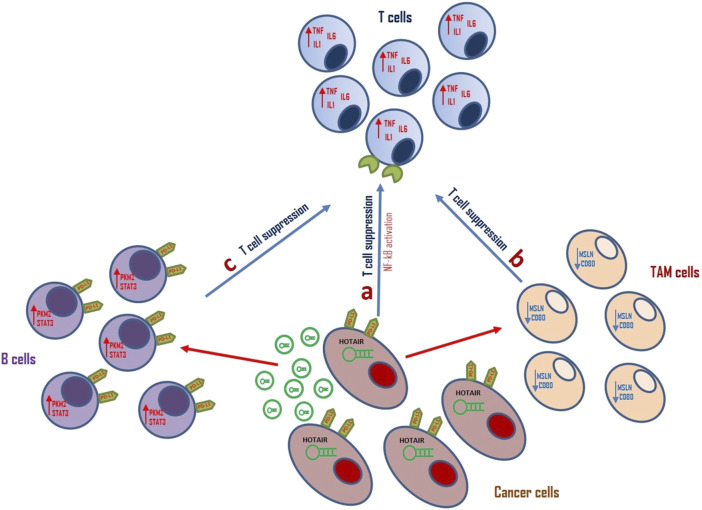
Schematic representation of the role of HOTAIR in tumor immune escape. **(A)** HOTAIR can induce PD-L1 expression on cancer cells surface. It can induce NF-kB pathway promoting T-cell suppression, thereby modulating tumor immune escape in glioma cells. **(B)** Aberrant HOTAIR expression can modulate TAM cell activity by downregulation of MSLN and CD80, thereby inducing T-cell suppression in LSCC TME. **(C)** Exosomal HOTAIR upregulates PDL1 expression on B cells surface promoting STAT3 expression and modulating tumor escape by CD8^+^ T cells suppression in CRC TME.

HOTAIR plays a critical role in the immune response during inflammation, being able to regulate the expression of proinflammatory cytokines, glucose transporter, and glucose metabolism in macrophages by activation of NF-κB pathway (Obaid et al., 2018; 2021). Although the initial evidence suggests that HOTAIR may play a role in immune escape (Figure 2), its function in resistance to immunotherapies has yet to be established.

Wang et al. showed that the expression of HOTAIR positively correlated with the mesenchymal subtype of glioma and negatively with the proneural subtype (Wang et al., 2021). In these cells, PD-L1 is positively regulated by HOTAIR and its activation can alter T cell toxicity through the NF-κB signalling pathway promoting inflammatory signalling and immune escape in glioma cells. The *in vitro* and *in vivo* inhibition of HOTAIR function blocked the expression of PD-L1 on the surface of glioma cells, promoting the infiltration of tumour-related immune cells (Wang et al., 2021).

HOTAIR can regulate PD-L1 expression also in LSCC. Yuan et aldescribed that overexpression of hsa-miR-30a-5p significantly inhibited PD-L1 levels in LSCC cells and this effect is restored by HOTAIR upregulation. These data suggested that HOTAIR can promote immune escape of LSCC cells through the modulation of ha-miR-30a-5p/GRP78/PD-L1 signalling (Yuan, Shen and Ma, 2022). Moreover, HOTAIR together with the lncRNA Metastasis Associated Lung Adenocarcinoma Transcript 1 (MALAT1) is involved in regulating oncogenic immune-modulatory proteins Mesothelin (MSLN) and CD80 in TAMs of HER2+ and TNBC subtypes of BC. This suggests that inhibiting HOTAIR and MALAT1 may affect the anti-inflammatory activity of tumour-associated macrophages in these tumours (Amer, Eissa and El Tayebi, 2022).

Xie and colleagues conducted a study examining the levels of HOTAIR in exosomes originating from colorectal cancer cells and in B cells that infiltrate the tumor.

Recently, Xie and colleagues executed a study examining the expression levels of HOTAIR in both CRC-derived exosomes and infiltrating B cells. They showed that HOTAIR is secreted by CRC cells through exosomes and it is upregulated in CRC-enriched B cells. Exosomal HOTAIR upregulated PDL1 expression on B cells thereby suppressing CD8^+^ T cell activity. HOTAIR can increase pyruvate kinase M2 (PKM2) protein expression in B cells protecting PKM2 protein from ubiquitination degradation and promoting STAT3 activation (Xie et al., 2023).

Although there is still no evidence of a role for HOTAIR in the immunotherapy response, its crucial role in the immune modulation could open a new scenario for understanding the mechanisms underlying immunotherapy resistance. Targeted studies on large series of patients should be performed to analyze the potential relationships between the aberrant expression of HOTAIR, also through liquid biopsy, and the response to immune checkpoint inhibitors.

## Conclusion

LncRNAs are increasingly recognized as key regulators in important biological processes involved in cancer progression, as well as playing a critical role in the development of resistance to cancer treatments.

Among these, HOTAIR has been abundantly described as a prognostic and predictive biomarker in most of solid and haematological tumours. Its aberrant activity, alone or by functional interaction with different miRNAs, in the modulation of drug resistance mechanisms has been reported. Numerous studies have highlighted the ability of HOTAIR to modulate the sensitivity of tumour cells in particular to cisplatin, although HOTAIR also appears capable of enhancing paclitaxel and doxorubicin resistance in different solid tumours. Indeed, its silencing can restore sensitivity to these drugs mainly by reactivating mechanisms related to autophagy and promoting apoptosis. Likewise, different studies showed that HOTAIR can modulate sensitivity and resistance to ionizing radiation especially by promoting hypoxia in addition to interacting with several miRNAs implicated in the activation of autophagy regulators. Furthermore, HOTAIR is involved in endocrine therapy resistance, especially in BC, in which its aberrant activity is induced both *in vitro* and *in vivo* by estrogens (Bhan and Mandal, 2016). Finally, new findings also indicate that HOTAIR may also play a role in modulating the response to multi-target tyrosine receptor kinase inhibitors through the interaction with the main signal transduction pathways that mediate cell growth and proliferation.

Due to its multiple functions in cancer initiation and progression, and its ability to influence sensitivity to anticancer drugs, it is suggested the possibility of using HOTAIR not only as a prognostic and predictive biomarker but also as a potential therapeutic target.

A series of small molecule compounds, such as AC1Q3QWB and AC1NOD4Q, functioning as HOTAIR-EZH2 inhibitors to block PRC2 recruitment, have been developed to suppress the activity of HOTAIR. For example, the use of AC1Q3QWB, by interrupting the HOTAIR-EZH2 interaction, enhances drug sensibility in endometrial cancer (L. Chen P. et al., 2023), while AC1NOD4Q showed the same effect on BC cells (Ren et al., 2019).

However, despite the numerous evidence on the role of HOTAIR as a diagnostic and prognostic marker in the majority of solid tumors, only one clinical trial performed on thyroid cancer patients (ClinicalTrials.gov Identifier: NCT03469544) is ongoing. Targeted studies should be conducted on large series of patients to validate its value as a biomarker since the detection of HOTAIR, both through molecular approaches and *in situ* analysis, in different biological matrices, could improve prognostic stratification and predict the sensitivity of tumor cells to different therapeutic strategies, representing a useful tool in the management of cancer patients.

## Figures and Tables

**Table:** 

**HOTAIR**	homeobox transcript antisense intergenic RNA
**si HOTAIR**	small interfering HOTAIR
**MDR**	multidrug resistance
**EMT**	epithelial-mesenchymal transition
**TME**	tumour microenvironment
**ncRNAs**	non-coding RNAs
**RNP**	ribonucleoprotein
**ABC**	ATP-binding cassette
**BCL-2**	B-cell lymphoma 2
**BAX**	pro-apoptotic proteins BCL2 associated X
**BAK**	BCL2 antagonist/killer 1
**FAS**	Fas cell surface death receptor
**AR**	androgen receptor
**PRC2**	Polycomb repressive complex
**LSD1**	lysine-specific histone demethylase 1A
**MTA2**	metastasis-associated gene 2
**PI3K**	Phosphoinositide 3-kinase
**BAD**	BCL2 associated agonist of cell death
**EZH2**	Enhancer of zeste 2 polycomb repressive complex 2 subunit
**MYC**	Avian Myelocytomatosis Viral Oncogene Homolog
**HSPA1A**	heat shock protein family A (Hsp70) member 1A
**HIF-1α**	Hypoxia-Inducible Factor-1alpha
**MMP**	metalloproteinase
**ATG12**	Autophagy Related 12
**MAPK1**	Mitogen-activated protein kinase 1
**JMJD6**	Jumonji domain-containing 6
**BRD4**	bromodomain containing 4
**Wnt3a**	wingless integrated 3a
**APC**	Adenomatous Polyposis Coli
**Oct**	octamer-binding transcription factor
**Sox2**	SRY-box transcription factor 2
**Klf4**	KLF transcription factor 4
**DNMT1**	DNA-methyltransferase 1
**H3K27me3**	H3 lysine 27 trimethylation
**PARP**	CI- Poli ADP-ribosio polimerasi
**NF-kB**	Nuclear factor kappa B
**VEGFA**	Vascular endothelial growth factor
**MRP1**	Multidrug resistance-associated protein 1
**GPC5**	glypican-5
**PTPN14**	Protein Tyrosine Phosphatase Non-Receptor Type 14
**CRNDE**	Colorectal Neoplasia Differentially Expressed
**UCA1**	Urothelial Cancer Associated 1
**SNAI2**	Snail Family Transcriptional Repressor 2
**CDKN1A**	cyclin-dependent kinase inhibitor 1A
**CALCOCO1**	Calcium Binding And Coiled-Coil Domain 1
**ZC3H10**	zinc finger CCCH-type containing 10
**STAT3**	Signal transducer and activator of transcription 3
**FOXA1**	Forkhead-box A1
**ATG2B**	autophagy-related protein 2 homolog B
**ALK**	anti-Anaplastic kinase lymphoma
**ROS1**	ROS Proto-Oncogene 1
**ULK1**	Unc-51 Like Autophagy Activating Kinase 1
**PD-1**	Programmed death 1
**PD-L1**	Programmed death ligand 1
**CTLA-4**	Cytotoxic T-Lymphocyte Antigen 4
**MALAT1**	Metastasis Associated Lung Adenocarcinoma Transcript 1
**MSLN**	Mesothelin
**PKM2**	protected pyruvate kinase M2
